# Analysis of the Cytoprotective Role of α-Crystallins in Cell Survival and Implication of the αA-Crystallin C-Terminal Extension Domain in Preventing Bax-Induced Apoptosis

**DOI:** 10.1371/journal.pone.0055372

**Published:** 2013-02-01

**Authors:** Séverine Hamann, Sylviane Métrailler, Daniel F. Schorderet, Sandra Cottet

**Affiliations:** 1 IRO, Institute for Research in Ophthalmology, Sion, Switzerland; 2 School of Life Sciences, Swiss Federal Institute of Technology (EPFL), Lausanne, Switzerland; 3 Department of Ophthalmology, University of Lausanne, Lausanne, Switzerland; UAE University, Faculty of Medicine & Health Sciences, United Arab Emirates

## Abstract

α-Crystallins, initially described as the major structural proteins of the lens, belong to the small heat shock protein family. Apart from their function as chaperones, α-crystallins are involved in the regulation of intracellular apoptotic signals. αA- and αB-crystallins have been shown to interfere with the mitochondrial apoptotic pathway triggering Bax pro-apoptotic activity and downstream activation of effector caspases. Differential regulation of α-crystallins has been observed in several eye diseases such as age-related macular degeneration and stress-induced and inherited retinal degenerations. Although the function of α-crystallins in healthy and diseased retina remains poorly understood, their altered expression in pathological conditions argue in favor of a role in cellular defensive response. In the *Rpe65^−/−^* mouse model of Leber's congenital amaurosis, we previously observed decreased expression of αA- and αB-crystallins during disease progression, which was correlated with Bax pro-death activity and photoreceptor apoptosis. In the present study, we demonstrated that α-crystallins interacted with pro-apoptotic Bax and displayed cytoprotective action against Bax-triggered apoptosis, as assessed by TUNEL and caspase assays. We further observed in staurosporine-treated photoreceptor-like 661W cells stably overexpressing αA- or αB-crystallin that Bax-dependent apoptosis and caspase activation were inhibited. Finally, we reported that the C-terminal extension domain of αA-crystallin was sufficient to provide protection against Bax-triggered apoptosis. Altogether, these data suggest that α-crystallins interfere with Bax-induced apoptosis in several cell types, including the cone-derived 661W cells. They further suggest that αA-crystallin-derived peptides might be sufficient to promote cytoprotective action in response to apoptotic cell death.

## Introduction

α-Crystallins, the major structural proteins of the mammalian lens, encompass αA- and αB-crystallins, which are encoded by separate genes [1]. The two α-crystallins have molecular masses around 20 kDa each and share 55% amino acid identity. Their molecular structure is similar, containing three distinct domains: a highly conserved central α-crystallin domain of around 90 amino acids, flanked by a variable hydrophobic N-terminal domain and a hydrophilic C-terminal extension containing a conserved sequence motif [2]–[4]. α-Crystallins belong to the small heat shock protein family of molecular ATP-independent chaperones. In mature lens fiber cells, they binds improperly folded proteins thereby preventing subsequent formation of light scattering aggregates [5]. Interactions between α-crystallins and putative substrates involve exposure of hydrophobic surfaces. However, emerging data support the idea that many sites may contribute to substrate interactions and that binding may be different according to the nature of the substrates [4], [6].

Besides their chaperone-like activity [1], [7], α-crystallins play a critical role in modulating various cellular processes such as oxidative stress, neuroprotection and apoptosis pathways, either promoting survival or inhibiting cell death [8]. In human lens-derived epithelial cell line, α-crystallins interfere with UVA-induced apoptosis through different mechanisms, including PKCα, Raf/MEK/ERK and Akt signaling pathways. While αB-crystallin is able to abrogate apoptosis through repression of Raf/MEK/ERK signal, αA-crystallin activates the Akt surviving pathway to inhibit triggered apoptosis [9]. In addition, αA-crystallin has been shown to inhibit apoptosis by enhancing phosphoinositide 3 kinase (PI3K) activity, which was related to its chaperone activity [10]. It has been observed that α-crystallins counteract the mitochondrial apoptotic pathway triggering the translocation of Bax at the mitochondria, the release of mitochondrial cytochrome C in the cytosol and the subsequent activation of downstream caspases including Caspase-3 [11]. In lens epithelial cells, interaction of α-crystallins with pro-apoptotic Bcl-2-related proteins and Caspase-3 prevents Bax and Bcl-X_S_ mitochondrial translocation and caspase activation [12], [13]. They display cytoprotective action against staurosporine (STS)- and UVA-induced apoptosis [14], [15], [9]. α-Crystallins protect cells from metabolic stress [16] as well as apoptosis induced by various stress factors such as STS [15], [17], TNF [15], [18], calcium [19], and hydrogen peroxide [20], [21]. αB-crystallin can inhibit apoptosis induced by TRAIL [22], DNA-damaging agent and growth factor deprivation [23], [24].

Microarray and proteome expression studies highlighted that αA- and αB-crystallins are expressed in normal and pathological retina [25]–[27]. Both proteins are detected in the ganglion cell layer as well as in the outer and inner nuclear layers of the retina [25]. During the course of retinal degeneration, α-crystallin expression is impaired in inherited retinal diseases in RCS rat [28], [29] and rd mouse [27], [30], after ischemia-reperfusion injury [31], following exposure to light injury [32], and in age-related macular degeneration (ARMD) [33]. Altered regulation of α-crystallins in ocular pathologies suggests that they may impact on the outcome of the related diseases. Disruption of αA-crystallin accentuates photoreceptor apoptosis and retinal degeneration in chemically-induced hypoxia [34] and in experimental uveitis [35], [36]. The current observations argue in favor of α-crystallins as part of a cellular protective response to the stress of the diseased retina.

We previously reported that the altered regulation of α-crystallins was correlated with triggering of the Bcl-2-apoptotic pathway during progression of the disease in the *Rpe65^−/−^* mouse model of Leber's congenital amaurosis (LCA), an autosomal recessive form of retinitis pigmentosa (RP) [37]. This decrease was correlated with mitochondrial translocation of pro-apoptotic Bax and photoreceptor apoptosis [38]. We further demonstrated the direct role of pro-apoptotic Bax in the apoptosis of rod photoreceptors in early [39] and late [40] stages of the disease. In the present study, we proposed to analyze the anti-apoptotic function of α-crystallins against Bax-mediated apoptosis. We further assessed which domain of αA-crystallin was involved in protecting against Bax-triggered apoptosis.

## Materials and Methods

### Cloning and Plasmids

The mouse αA (αA)- and αB (αB)-crystallin cDNAs were amplified by RT-PCR from mouse retina mRNA using the following primers: 5′-ATGGACGTCACCATTCAGCATCCTTGGTTCAAGCGTGCCCTGG-3′ (αA-for), 5′-TCAGGACGAGGGTGCAGAGCTG-3′ (αA-rev), 5′-ATGGACATCGCCATCCACCACCCCTGGATCCGGCGCCCCTTC-3′ (αB-for), 5′-CTACTTCTTAGGGGCTGCGGCG-3′ (αB-rev). The cDNAs were then inserted into pGEM-T cloning vector (pGEM®-T Easy Vector Systems; Promega, Dübendorf, Switzerland). NotI-digested αA- and αB-crystallin inserts from pGEM-T constructs were further subcloned into pcDNA3.1 expression vector (pcDNA3.1-αA/αB) at the NotI site. The pRluc-αA-crystallin and pRluc-αB-crystallin fusion constructs were created by PCR and fused in frame at the N-terminus of luciferase (pRluc-N2 vector) at the BglII and XhoI sites, using the following primers:

5′-gatcagatctgccaccatggacgtcaccattcag-3′ (BglII-αA-for), 5′-gatcctcgagggacgagggtgcagagc-3′ (XhoI-αA-rev);

5′-gatcagatctgccaccatggacatcgccatccac-3′ (BglII-αB-for), 5′-gatcctcgagcttcttaggggctgcggc-3′ (XhoI-αB-rev).

The pRluc-αA-crystallin mutant constructs were generated in the same way using the following primers:

αA_1-116: 5′-GATCAGATCTGCCACCATGGACGTCACCATTCAG-3′ (BglII-αA-for), 5′-GATCCTCGAGACGGTGAAATTCAC-3′ (XhoI-αA_1-116-rev);

αA_117-173: 5′-GATCAGATCTGCCACCATGCGCTACCGTCTG-3′ (BglII-αA_117-173-for), 5′-GATCCTCGAGGGACGAGGGTGCAGAGC-3′ (XhoI-αA-rev);

αA_1-89: 5′-GATCAGATCTGCCACCATGGACGTCACCATTCAG-3′ (BglII-αA-for), 5′-GATCCTCGAGTACCTTCACGGTGAGGTC-3′ (XhoI-αA_1-89-rev);

αA_90-143: 5′-GATCAGATCTGCCACCATGCTGGAGGATTTTGTGGAG-3′ (BglII-αA_90-143-for), 5′-GATCCTCGAGGCCAGAGAAGGTCAGCATG-3′ (XhoI-αA_90-143-rev);

αA_144-173: 5′-GATCAGATCTGCCACCATGCCCAAGGTCCAGTCC-3′ (BglII-αA_144-173-for), 5′-GATCCTCGAGGGACGAGGGTGCAGAGC-3′ (XhoI-αA-rev);

αA_64-143: 5′-GATCAGATCTGCCACCATGGTCCGATCTGAC-3′ (BglII-αA_64-143-for), 5′-GATCCTCGAGGCCAGAGAAGGTCAGCATG-3′ (XhoI-αA_64-143-rev).

The bicistronic lentiviral vector pWPI (www.addgene.org, Addgene plasmid 12254), generously provided by Dr. D. Trono (Ecole Polytechnique Fédérale de Lausanne (EPFL), Lausanne, Switzerland), was used to subclone PCR-amplified αA-myc and αB-myc inserts at the PacI site (pWPI-αA and pWPI-αB) using the following primers: 5′-GATCTTAATTAAGCCACCATGGACGTCACCATTCAG-3′ (PacI-αA-for), 5′-GATCTTAATTAATCACAGATCTTCTTCAGAAATAAGTTTTTGTTCGGACGAGGGTGCAGAGCTGG-3′ (PacI-αA-myc-rev), 5′-GATCTTAATTAAGCCACCATGGACATCGCCATCCAC-3′ (PacI-αB-for), 5′-GATCTTAATTAATCACAGATCTTCTTCAGAAATAAGTTTTTGTTCCTTCTTAGGGGCTGCGGCG-3′ (PacI-αB-myc-rev). pcDNA3-Bax plasmid was generously provided by Dr. S. Matsuyama (Case Western Reserve University, Cleveland, USA).

### Cell culture and transient transfection

Embryonic kidney (HEK) 293T cells were grown in Dulbecco's modified Eagle's medium (DMEM) (PAA Laboratories E15-883, Pasching, Austria) supplemented with 25 mM Hepes, 10% FBS (Biowhittaker® DE14-802F, Lonza Verviers SPRL, Verviers, Belgium), 100 U/ml penicillin and 100 µg/ml streptomycin (Life Technologies Europe/GIBCO, Zoug, Switzerland). Mouse photoreceptor-derived 661W cell line was grown in DMEM (PAA Laboratories E15-883) supplemented with 25 mM Hepes, 1 mM sodium pyruvate, 10% FBS (Biowhittaker® DE14-802F), 0.6 mM ß-mercaptoethanol (Applichem, Darmstadt, Germany), 100 U/ml penicillin and 100 µg/ml streptomycin (Life Technologies). 661W cells were generously provided by Dr. M. Al-Ubaidi (University of Oklahoma, Olkahoma City, USA) [41].

293T cells were transiently transfected with the calcium phosphate method (ProFection®, Promega) or with the cationic polymer method (jetPEI™, Polyplus-transfection, Illkirch, France). Cells were transfected with the following total amount of plasmids: 3.5 µg and 2 µg using ProFection® and jetPEI™, respectively, in 12-well plates (TPP, Trasadingen, Switzerland), 0.25 µg using jetPEI™ in 96-well plates (TPP) and 8 µg using jetPEI™ in p100 plates (TPP). 661W cells in p100 plates were transiently transfected with 24 µg of plasmids using the cationic lipid method (Lipofectamine LTX®/PLUS™, Life Technologies). To keep the total amount of transfected DNA constant, appropriate quantities of empty plasmids were added in all experiments. All plasmids were prepared on NucleoBond® PC500 columns (Macherey-Nagel, Düren, Germany).

### Preparation of lentiviral vectors and transduction of 661W cells

Approximately 1000-fold concentrated, high titer stocks of lentiviral vectors packaged by the multiply attenuated lentivirus pCMVDR8.74 and pseudotyped with the vesicular stomatitis virus-G (VSV-G) envelope protein (plasmid pMD2.G) were obtained by transient co-transfection of 293T cells with the corresponding lentiviral expression vectors (pWPI, pWPI-αA and pWPI-αB), as previously described [42], [43]. The pWPI bicistronic vector allows for simultaneous expression of a transgene and GFP fluorescent marker, the latter being inserted downstream of an internal ribosome entry site from encephalomyocarditis virus (IRES-EMCV). Approximately 90–95% of the 661W cells were transduced with the recombinant lentiviruses, according to GFP fluorescence tracking, and stably expressed the target genes as assessed by immunofluorescence and western blotting.

### Co-immunoprecipitation

Cleaning of the antibody used for co-immunoprecipitation (co-IP) was done using the Melon Gel IgG purification support to remove gelatin from the IgG sample (Pierce® Antibody Clean-up kit; Thermo Fisher Scientific, Lausanne, Switzerland). Co-IP was performed according to manufacturer's instructions (Pierce® Co-Immunoprecipitation kit; Thermo Fisher Scientific). Briefly, 293T cells grown in p100 plates were lysed in 500 µl IP lysis/wash buffer containing freshly added Protease inhibitor cocktail tablets (Roche, Rotkreuz, Switzerland). 1000 to 2000 µg of total proteins in 500 µl final volume IP lysis/wash buffer were immunoprecipitated with 15 to 30 µg of pre-cleaned rabbit anti-Bax antibody (sc-493; Santa Cruz Biotechnology, Heidelberg, Germany) coupled to AminoLink® Plus Coupling Resin overnight at 4°C on a rotary wheel, followed by 3 washes in IP lysis/wash buffer. The immunoprecipitated complex was then eluted in 30 to 60 µl of elution buffer. Immunoprecipitated samples and whole cell extracts were resolved on 12% SDS-PAGE and transferred on PVDF membranes (Whatman/Schleicher & Schuell, Sanford, United Kingdom). Membranes were blocked in 5% non-fat dried milk before being immunoassayed to detect α-crystallins and Bax, using mouse monoclonal anti-myc (diluted 1/10'000, from the Protein Expression Core Facility, EPFL, Lausanne, Switzerland) or rabbit polyclonal anti-αA/αB-crystallin (diluted 1/1'000, ADI-SPA-224; Enzo Life Sciences, Lausen, Switzerland) and rabbit polyclonal anti-Bax (diluted 1/2'000, sc-493; Santa Cruz) antibodies, respectively.

### Western blot analysis

Cells in p100 plates were lysed in 200 µl RIPA buffer containing freshly added Protease inhibitor cocktail tablets (Roche). Equivalent amounts of protein, as determined by the colorimetric Bradford protein assay (Bio-Rad Laboratories AG, Reinbach, Switzerland), were resolved on 12% SDS-PAGE followed by transfer on PVDF membrane (Whatman/Schleicher & Schuell). Membranes were blocked in 5% non-fat dried milk before being immunoassayed using mouse monoclonal antibodies directed against myc (diluted 1/10'000; EPFL), ß-actin (diluted 1/10'000, A5441; Sigma-Aldrich Chemie, Buchs, Switzerland) and luciferase (diluted 1/1'000, L6003-20; USBiological, Swampscott, USA), and rabbit polyclonal antibodies directed against GFP (diluted 1/5'000, G1544; Sigma) and αA/αB-crystallin (diluted 1/1'000, ADI-SPA-224; Enzo Life Sciences).

### Caspase assays

Caspase-Glo assay (Caspase-Glo® 3/7 Assay, Promega) was performed according to manufacturer's instruction. Briefly, 293T cells in 96-well plate (7×10^3^ cells/well) were incubated in 100 µl/well of Caspase-Glo® 3/7 Reagent for 30 min at room temperature (RT) in the dark before measuring the luminescent signal in a plate-reading luminometer as directed by the luminometer manufacturer. Caspase colorimetric assay (BioVision, Milpitas, USA) was performed according to manufacturer's instruction. Briefly, 100 to 200 µg of proteins from cell extracts were incubated in 200 µM of Caspase-3/-7-specific DEVD-*p*NA substrate for 1 h at 37°C. Spectrophotometric detection of the chromophore *p*-nitroanilide (*p*NA) liberated after caspase cleavage was quantified using a microtiter plate reader at 400-/405-nm.

### Terminal dUTP Nick End-Labeling (TUNEL) of fragmented DNA

DNA strand breaks in cell nuclei were detected by TUNEL assay, accroding to manufacturer's instruction. Briefly, cells grown on 0.1% gelatin-coated glass coverslips were fixed in 4% paraformaldehyde (PFA)/phosphate-buffered saline (PBS) for 20 min at RT, permeabilized in 0.1% Triton X-100/0.1% sodium citrate for 2 min on ice and incubated with terminal deoxynucleotidyl transferase (TdT) and fluorescein-12-dUTP or TMR-dUTP for 1 h at 37°C. Cells were also counterstained with 4′,6-diamidino-2-phenylindole, dihydrochloride (DAPI; Life Technologies) or Hoechst 33342 (Sigma) to identify cell nuclei. Following 3 washes in PBS, coverslips were mounted in Citifluor AF1 (Citifluor, London, United Kingdom) and viewed under a fluorescence microscope (Olympus BX61) using appropriate filters. For each condition, the number of TUNEL-positive apoptotic cells relative to the number of DAPI-stained viable cells was counted in three areas of the plated cells and the resulting numbers from each experiment (n = 2–3) were averaged.

### Luminescence ATP detection assay

Measure of cellular ATP content (ATPLite™, PerkinElmer) was performed to assess cell viability following STS (Sigma) treatment, according to manufacturer's instruction. Briefly, 661W cells in 96-well plate (7×10^3^ cells/well) were incubated in 50 µl/well of mammalian cell lysis solution and 50 µl/well of substrate solution, followed by 10 min incubation at RT in the dark before measuring the luminescence in a plate-reading luminometer.

### Immunofluorescence

Cells grown on 0.1% gelatin-coated coverslips were fixed in 4% PFA/PBS for 20 min at RT, washed briefly with PBS and permeabilized in 0.2% Triton X-100/PBS for 2 min. Cells were then blocked in PBS with 10% normal goat serum (NGS, G9023; Sigma) and 0.2% Triton X-100 (Sigma) for 1 h at RT. Immunodetection was performed by incubation with primary antibodies in PBS with 2% NGS and 0.2% Triton X-100 overnight at 4°C, followed by incubation with fluorochrome-conjugated secondary antibody for 1 h at RT. Incubation with non-immune immunoglobulin fraction (Sigma) of the same species as the primary antibody was used as a negative control. Species and dilutions of the antibodies used were as follows: rabbit anti-αA/αB-crystallin (diluted 1/500, ADI-SPA-224; Enzo Life Sciences, Lausen, Switzerland), mouse anti-myc (diluted 1/1'000; EPFL), mouse anti-luciferase (diluted 1/100, L6003-20; USBiological) and Alexa Fluor 594 goat anti-mouse IgG (diluted 1/1'000, A11005; Life Technologies). Following 3 washes in PBS, coverslips were mounted in Citifluor AF1 (Citifluor). Cells were counterstained with DAPI (Life Technologies) to identity cell nuclei.

### Imaging

Images were viewed under a fluorescence microscope equipped with a digital camera (Olympus BX61; Olympus, Lausanne, Switzerland) using appropriate filters.

### Statistical analysis

All results were expressed as means ± SEM of the indicated number of experiments. Statistical significance was calculated with the *t*-test.

## Results

### Anti-apoptotic activity of α-crystallins against Bax-induced cell death

**Figure 1:**
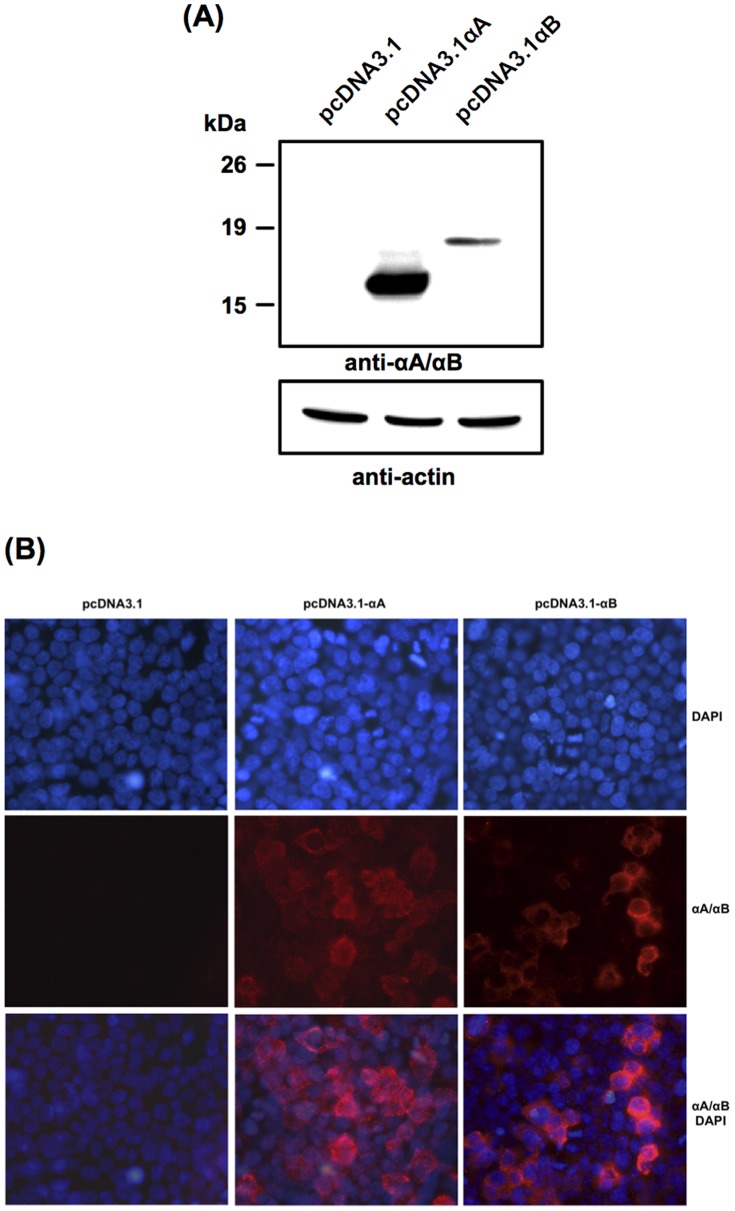
Expression of αA- and αB-crystallins in transiently transfected 293T cells. (**A**) Western blot analysis of αA- and αB-crystallin levels 24 h post-transfection. Fifty micrograms of total proteins from cell extracts were subjected to 12% SDS-PAGE and immunoassayed with anti-αA/αB-crystallin to detect the overexpressed α-crystallins and with anti-ß-actin as a control of equal protein loading. (**B**) Immunofluorescence analysis with anti-αA/αB-crystallin showing cytoplasmic expression of αA- (pcDNA3.1-αA) and αB- (pcDNA3.1-αB) crystallins 24 h post-transfection, while no detection was observed in cells transfected with the empty plasmid (pcDNA3.1).

To evaluate the cytoprotective action of α-crystallins, we first cloned both αA- and αB-crystallin cDNAs from mouse retina. Protein expression was then assessed in 293T cells transfected for 24 h with either αA- or αB-crystallin. As shown by western blot analysis, αA- and αB-crystallin proteins of the expected size were detected in cells transiently transfected with pcDNA3.1-αA and pcDNA3.1-αB constructs, respectively, while no protein was observed in cells transfected with the empty pcDNA3.1 plasmid (Fig. 1A). The cytoplasmic localization of the overexpressed αA- and αB-crystallins was further observed by immunofluorescence (Fig. 1B).

**Figure 2:**
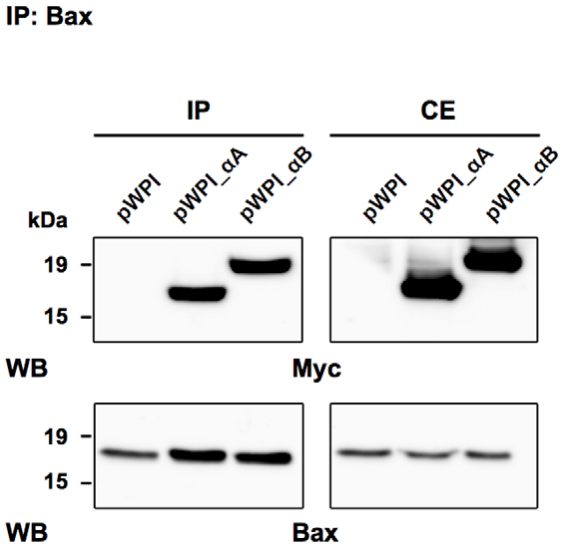
Interaction of α-crystallins with Bax *in vivo*. 293T cells transiently transfected with the empty vector (pWPI), myc-tagged αA- (pWPI_aA) or αB- (pWPI_aB) crystallin were further treated with 100 nM STS for 3 h before co-immunoprecipitation with anti-Bax antibody. The precipitated samples were then sequentially probed by western blot using anti-myc and anti-Bax antibodies. IP: immunoprecipitated samples (*left panels*); CE: 20 µg of total proteins from whole cell extract (*right panels*).

**Figure 3:**
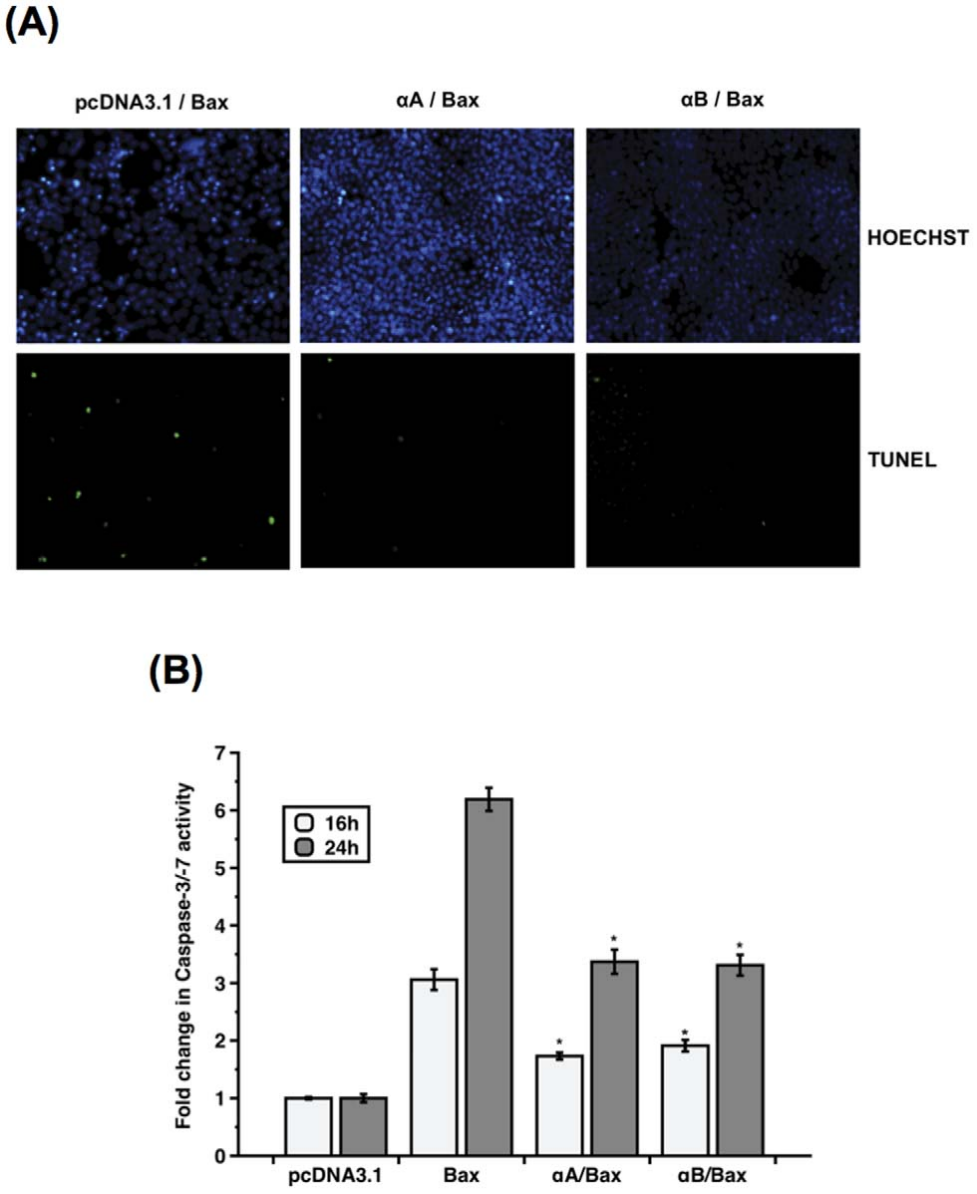
Anti-apoptotic activity of α-crystallins against Bax-induced apoptosis. 293T cells were transiently co-transfected with pcDNA3-Bax and with either the empty pcDNA3.1, pcDNA3.1-αA- (αA) or pcDNA3.1-αB (αB)-crystallin constructs. (**A**) TUNEL assay showing that Bax-triggered apoptosis was inhibited in 293T cells overexpressing the α-crystallins, as reflected by the reduced number of TUNEL-positive apoptotic cells. Cell nuclei were counterstained with Hoechst. (**B**) As assessed by luminescent caspase assay, Bax-induced Caspase-3/-7 activity was inhibited in the presence of αA- (αA) and αB- (αB) crystallins 16 h and 24 h post-transfection. (* p<0.0001 by *t*-test for Bax *versus* αA/Bax and for Bax *versus* αB/Bax at 16 h and 24 h). Data are the mean ± SE of three independent experiments, each performed in triplicates.

It has been shown in lens-derived epithelial cells that αA- and αB-crystallins interacted with pro-apoptotic Bax and prevented stress-induced apoptosis [13]. We thus investigated the interaction of α-crystallins and Bax *in vivo* in 293T cells overexpressing αA- or αB-crystallin. Cells were treated with 100 nM STS for 3 h before performing co-immunoprecipitation to assess the interaction of endogenous Bax with α-crystallins. Binding of Bax with both αA- and αB-crystallins was confirmed in cells transfected with the lentiviral vector expressing myc-tagged αA- (pWPI_αA) or αB- (pWPI_αB) crystallin, whereas no protein was co-immunoprecipitated in cells transfected with the empty vector (pWIP) (Fig. 2). The anti-apoptotic action of α-crystallins against Bax-induced apoptosis was then assessed by caspase and TUNEL assays in 293T cells overexpressing α-crystallins and Bax. 293T cells were initially transfected for 24 h with the empty plasmid (pcDNA3.1), pcDNA3.1-αA-crystallin (αA) or pcDNA3.1-αB-crystallin (αB) constructs, before to be co-transfected for 24 h with Bax. Forty-eight hours post-transfection, TUNEL assay was performed using fluorescein-12-dUTP to detect TUNEL-positive apoptotic cells. As shown in Fig. 3A, dense green fluorescence staining of apoptotic nuclei was observed in cells overexpressing Bax, whereas Bax-triggered apoptosis was inhibited in the presence of either αA- or αB-crystallin. The cytoprotective activity of α-crystallins against Bax-triggered apoptosis was further confirmed in co-transfected 293T cells, by measuring Caspase-3/-7 activity using a luminogenic substrate containing the Caspase-3/-7-specific DEVD amino acid sequence. Caspase activity was induced 3- to 6-fold in cells overexpressing Bax 16 h and 24 h post-transfection, respectively. However, Bax-induced caspase activity was inhibited by around 50% in the presence of either αA- (αA) or αB- (αB) crystallin (Fig. 3B).

### Staurosporine induced apoptosis in 661W cells

The cone-derived photoreceptor cell line 661W was initially isolated from a mouse retina transformed with the SV40 T-antigen under the control of the human interphotoreceptor retinol-binding protein (IRBP) promoter [41]. These cells express cone-specific markers including blue and green opsins, transducin (*Gnat2*) and arrestin (*Arr3*) [44].

**Figure 4:**
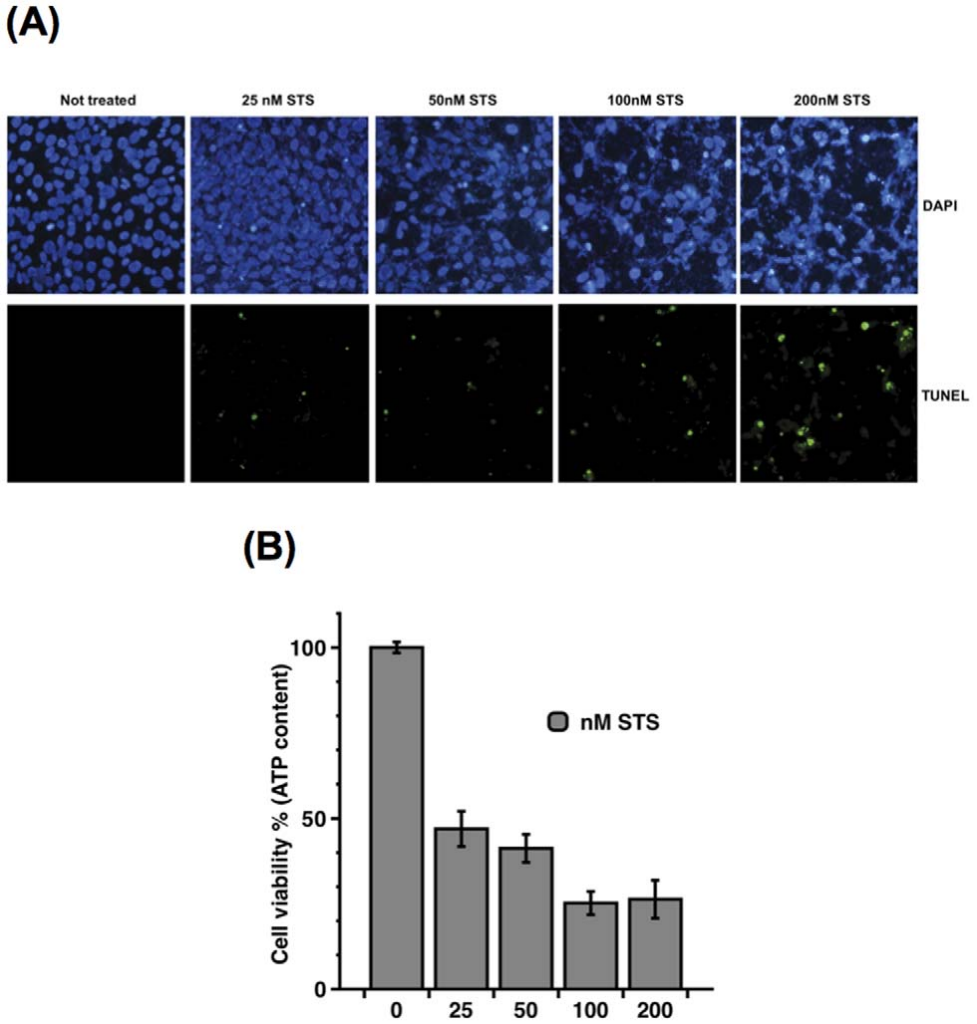
STS-induced apoptosis in 661W cells. Dose-dependent induction of apoptosis in 661W cells exposed to increasing amounts of STS (25 to 200 nM) for 24 h, as depicted by (**A**) increased TUNEL-positive apoptotic cells and (**B**) decreased level of intracellular ATP content. Data are the mean ±SE of at least four independent experiments, each performed in triplicates.

Staurosporine (STS), a protein kinase C inhibitor, preferentially activates the mitochondrial apoptotic pathway relying on Bax and caspase activation [11], [45], [46]. The effect of STS on 661W cell viability was assessed following exposure to increasing concentrations of the drug for 24 h. Cell viability was then evaluated by TUNEL assay as well as by measuring cellular ATP content. As a marker of cell viability, ATP is present in all metabolically active cells and its intracellular concentration declines very rapidly when cells die. Upon STS treatment, apoptotic cell death was induced in a dose-dependent manner, as reflected by the increase in TUNEL-positive apoptotic cells from 25 to 200 nM STS (Fig. 4A). A massive reduction in cellular ATP content was observed in 661W cells exposed to 25 nM STS and was further decreased at the highest concentrations of the drug (Fig. 4B).

### α-Crystallins were stably expressed in 661W cells

**Figure 5:**
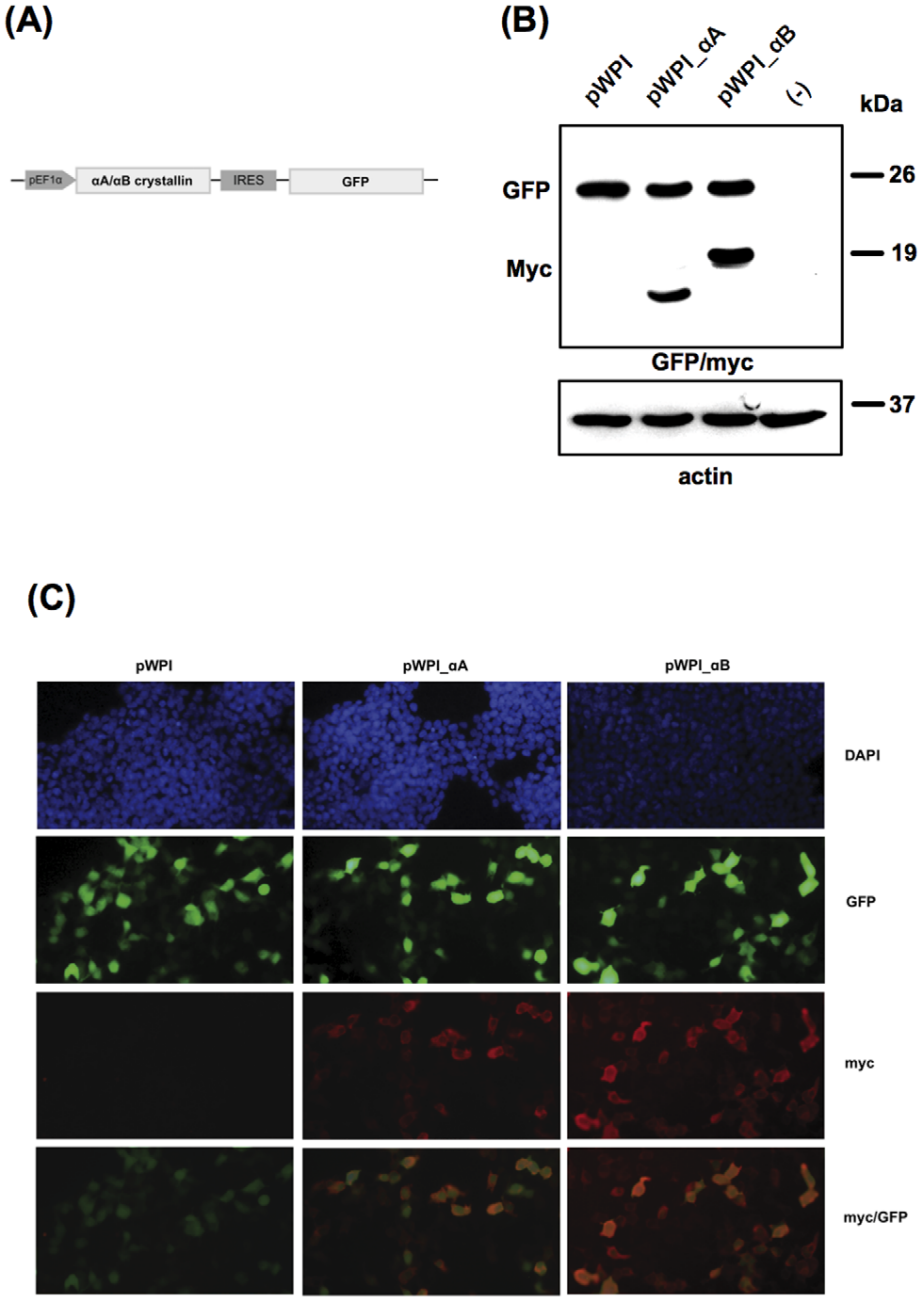
Stable expression of α-crystallins in 661W cells. (**A**) Schematic representation of the bicistronic pWPI lentiviral vector allowing for pEF1α-driven simultaneous expression of the transgene (αA- or αB-crystallin) and IRES-mediated GFP fluorescent marker. (**B**) Western blot analysis of αA- and αB-crystallins expressed in lentivirus-transduced 661W cells. Twenty-five micrograms of total proteins from cell extracts were subjected to 12% SDS-PAGE. Myc-tagged αA- and αB-crystallins were expressed in 661W cells transduced with the recombinant lentiviruses pWPI_αA and pWPI_αB, respectively, while no transgene was detected in cells transduced with the empty lentivirus pWPI or in non transduced cells (−). Membranes were further immunoassayed with anti-GFP as a control of transduction efficiency and with anti-ß-actin as a control of equal protein loading. (**C**) Immunofluorescence analysis with anti-myc showing cytoplasmic expression of αA- and αB-crystallins in 661W cells transduced with the corresponding recombinant lentiviruses. Cell nuclei were counterstained with DAPI and GFP staining was shown as a control of transduction efficiency. pEF1α: EF1α promoter; IRES: internal ribosome entry site from encephalomyocarditis virus.

To evaluate the anti-apoptotic activity of α-crystallins in photoreceptor-like 661W cells, we first generated stable cell lines overexpressing αA- or αB-crystallin. To achieve this, 661W cells were transduced with the recombinant lentiviruses overexpressing αA-crystallin (pWPI_αA) or αB-crystallin (pWPI_αB), or with the empty lentivirus (pWPI), and pools of transduced cells were expanded (Fig. 5A). As observed by western blot analysis, αA- and αB-crystallins were expressed in pWPI_αA- and pWPI_αB-transduced 661W cells, respectively, while no expression of the transgene was detected neither in cells transduced with the empty lentivirus nor in non transduced cells. As a control of transduction efficiency, all transduced cell lines expressed the GFP marker gene, while no protein was visible in non-transduced 661W cells (Fig. 5B). Immunofluorescence analysis showed that most of the cells were transduced with the recombinant lentiviruses, as reflected by GFP fluorescence and α-crystallin staining with anti-myc antibody. In addition, α-crystallins were essentially localized in the cytoplasm while GFP showed nuclear and cytoplasmic localization (Fig. 5C). Of note, clonal populations of cells overexpressed the transgenes with different levels of expression.

### STS-induced apoptosis was prevented in 661W cells in the presence of α-crystallins

**Figure 6:**
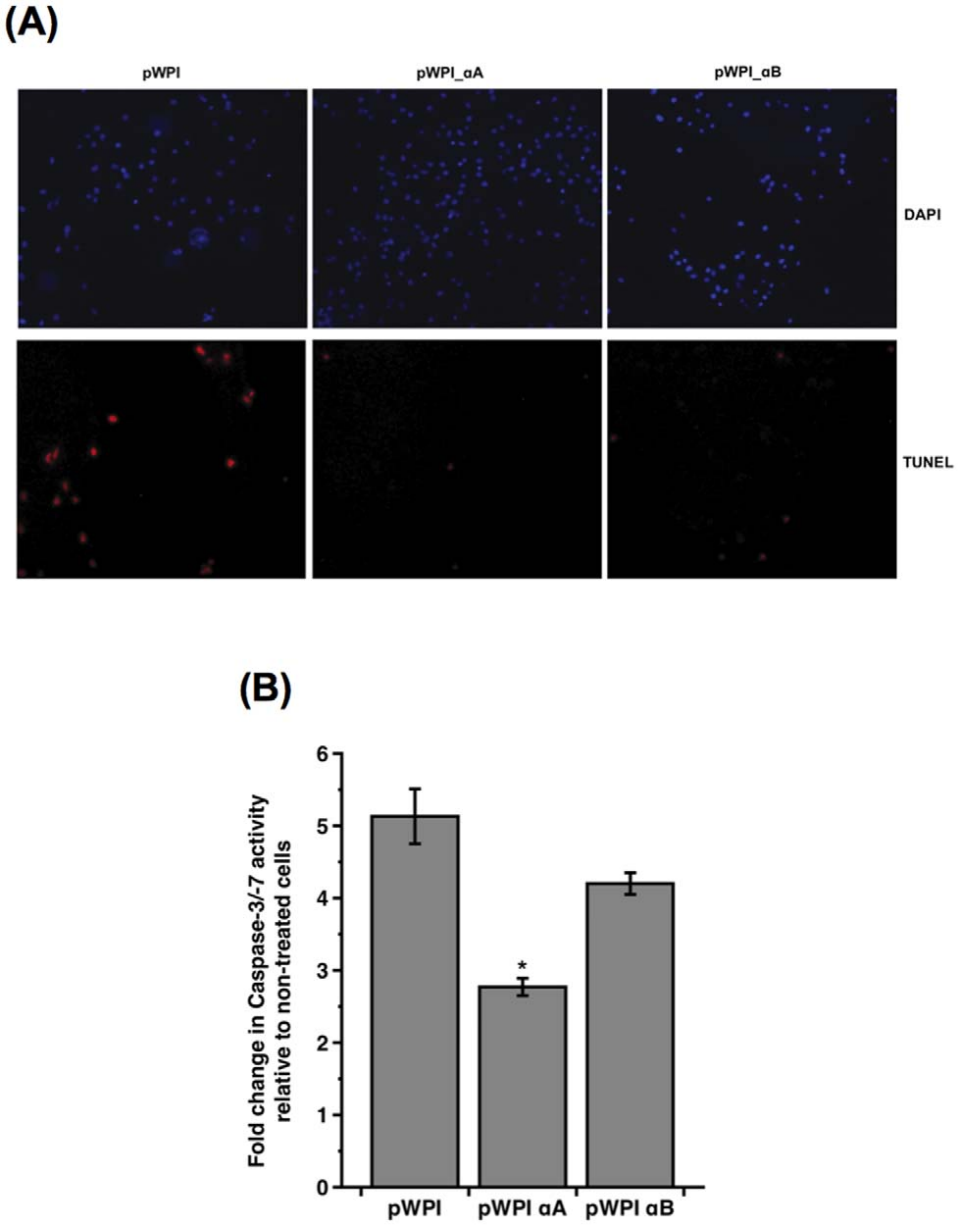
STS-induced apoptosis was prevented in 661W cells in the presence of α-Crystallins. 661W cells transduced with the recombinant lentiviruses overexpressing αA-crystallin (pWPI_αA), αB-crystallin (pWPI_αB) or the empty lentivirus (pWPI) were treated with 100 nM STS for 16 h. (**A**) STS-triggered apoptosis was inhibited in the presence of α-crystallins, as reflected by TUNEL assay using TMR-dUTP. (**B**) STS-induced caspase activation was decreased in 661W cells overexpressing αA- and αB-crystallins, as measured by colorimetric Caspase-3/-7 assay. (* p<0.005 by *t*-test for pWPI *versus* pWPIαA). Data are the mean ± SE of four independent experiments.

To further assess whether α-crystallins may counteract Bax-mediated apoptosis, 661W cells overexpressing αA- or αB-crystallin were exposed to 100 nM STS for 16 h. In TUNEL assay, using TMR-dUTP to detect DNA-strand breaks, STS-triggered apoptosis was markedly reduced in the presence of αA- and αB-crystallins, as compared with 661W cells transduced with the empty lentivirus (Fig. 6A). We then investigated whether α-crystallins may interfere with STS-induced activation of effector caspases using a Caspase-3/-7 colorimetric assay. Following exposure to STS, caspase activation was induced in 661W cells, as reflected by a 5-fold increase in Caspase-3/-7 activity in pWPI-transduced cells. However, caspase activity was inhibited in the presence of α-crystallins, with around 50% and 20% reduction in 661W cells overexpressing αA- and αB-crystallins, respectively (Fig. 6B).

### The C-terminal extension domain of αA-crystallin was sufficient to prevent Bax-induced apoptosis

**Figure 7:**
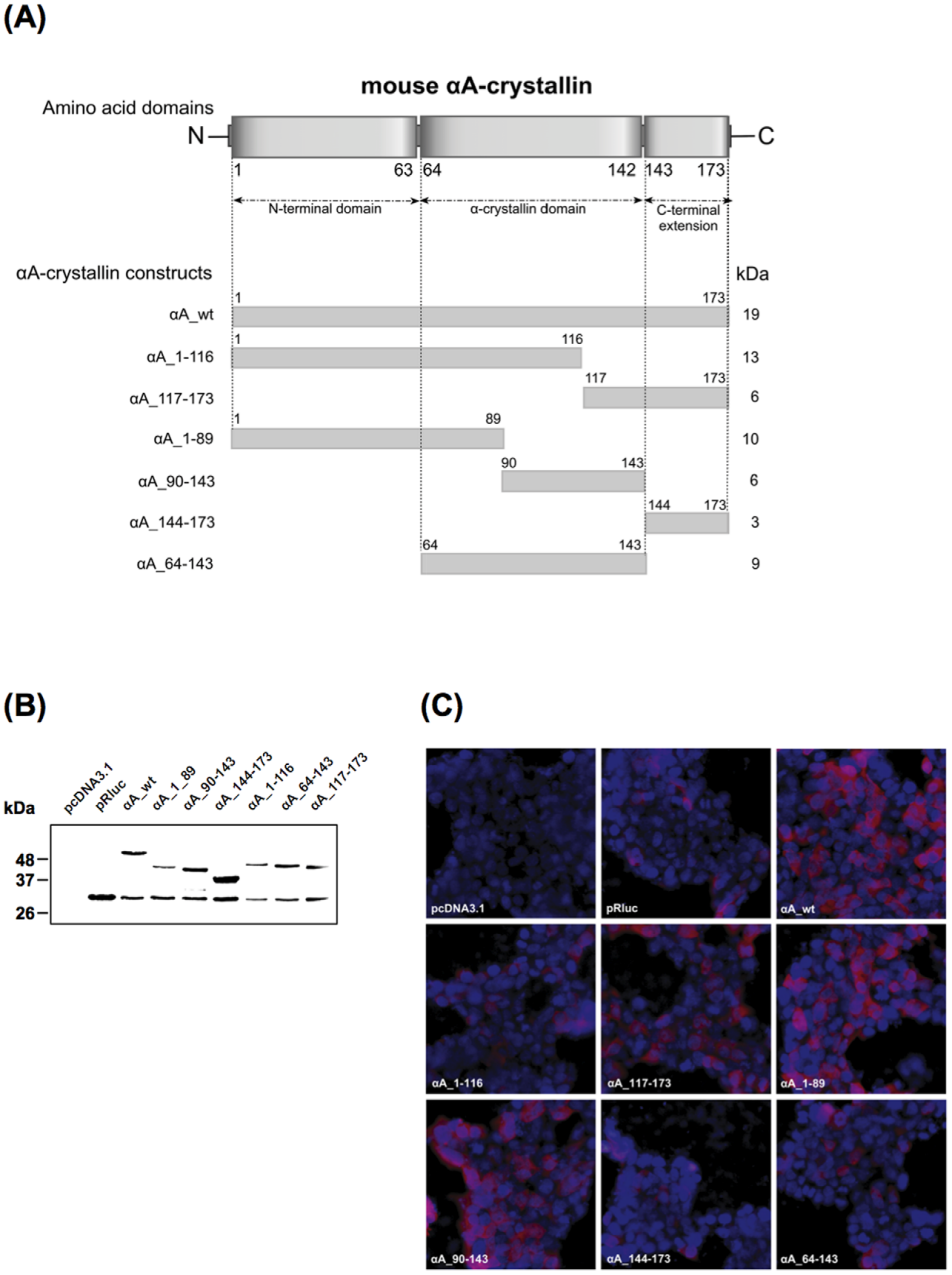
Generation and expression of αA-crystallin deletion mutants. (**A**) Schematic representation of the various deletion mutants of αA-crystallin. (**B**) Western blot and (**C**) immunofluorescence analyses in transiently transfected 293T cells showing expression of wt and mutant αA-crystallin proteins fused to luciferase using luciferase antibody.

α-Crystallins are characterized by a conserved α-crystallin domain flanked by a N-terminal domain and a short C-terminal extension [2]–[4]. To identify the domain of αA-crystallin sufficient to protect against Bax-induced apoptosis, we generated deletion mutants corresponding to distinct domains of αA-crystallin (Fig. 7A). Fusion proteins were created in which full-length and mutant αA-crystallins were fused in frame at the N-terminus of luciferase.

As 661W cells are low-efficient transfectable cells, we evaluated the anti-apoptotic properties of αA-crystallin mutants in 293T cells transiently transfected with the different constructs. Ectopic expression of full-length and mutant αA-crystallins was verified by western blot analysis (Fig. 7B). Bands of the expected size were detected for wt and mutant proteins. A 34-kDa band corresponding to luciferase was also observed in cells over-expressing the αA-crystallin proteins, as well as in cells transfected with the empty pRluc plasmid. This may be explained by translational leakiness as the pRluc plasmid contains an internal ATG start codon at the N-terminus of luciferase. As a control, no signal was detected in cells transfected with the pcDNA3.1 plasmid. Immunofluorescence analysis confirmed the cytoplasmic localization of the different mutants, similarly to wt αA-crystallin (Fig. 7C).

**Figure 8:**
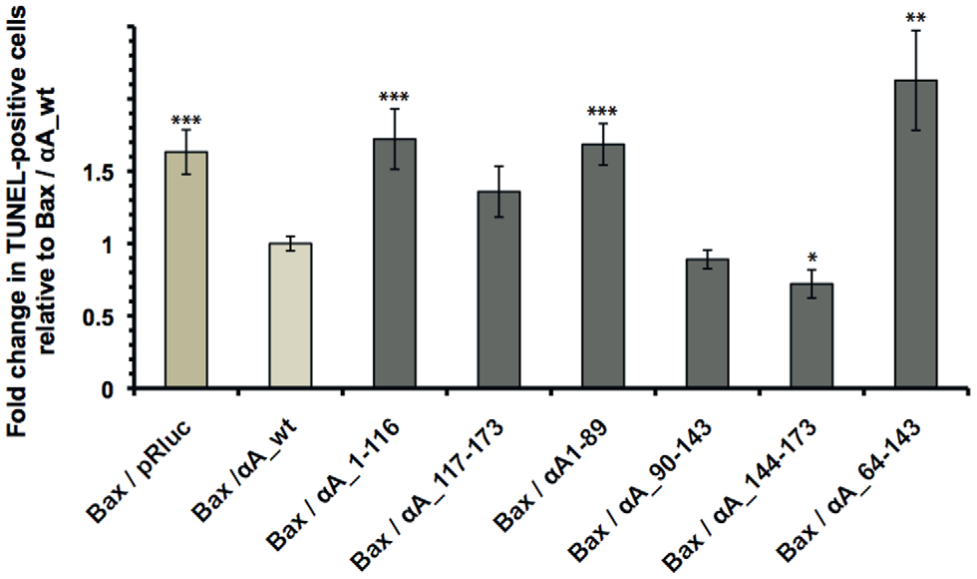
The C-terminal extension domain of αA-crystallin was sufficient to provide protection against Bax-induced apoptosis. Twenty-four hours post-transfection, 293T cells transfected with Bax (Bax/pRluc) or with Bax and αA-crystallin wt or mutants were assayed in TUNEL assay. Counting of TUNEL-positive apoptotic cells showed that αA_wt (Bax/αA_wt), αA_90-143 (Bax/αA_90-143) and αA_144-173 (Bax/αA_144-173) significantly inhibited Bax-induced apoptosis, whereas N-terminal αA_1-89 (Bax/αA_1-89) and αA_1-116 (Bax/αA_1-116), along with αA_64-143 (Bax/αA_64-143) containing the α-crystallin domain, did not prevent apoptosis. (* p<0.05, ** p<0.01, *** p<0.005 by *t*-test *versus* Bax/αA_wt). Data are the mean ± SE of two to three independent experiments.

The anti-apoptotic activity of the different αA-crystallin mutants against Bax-induced apoptosis was then assessed in 293T cells co-transfected with Bax and with wt or mutant αA-crystallins. Twenty-four hours post-transfection, TUNEL assay was performed to detect and count TUNEL-positive apoptotic cells. As shown in Fig. 8, wt αA-crystallin (Bax/αA_wt) inhibited Bax-triggered apoptosis (Bax/pRluc). Moreover, αA_90-143 mutant was as efficient as wt protein to prevent apoptosis, while the C-terminal extension αA_144-173 mutant significantly displayed better protection than the full-length αA-crystallin. However, we can not exclude that it may reflect differences in the levels of expression of the corresponding proteins. In addition, N-terminal αA_1-89 and αA_1-116 mutants, along with αA_64-143 mutant containing the α-crystallin domain, did not protect against Bax-induced apoptosis.

**Figure 9:**
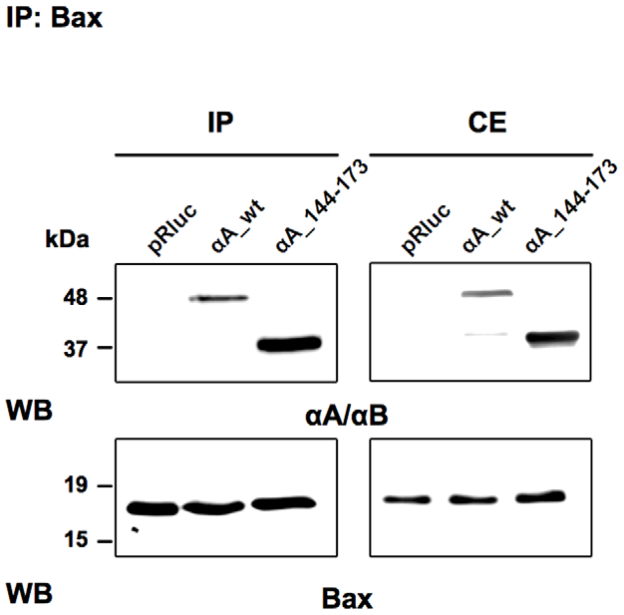
Interaction of the C-terminal extension domain of αA-crystallin with Bax *in vivo*. 293T cells transiently transfected with the empty vector (pRluc), full length αA- (αA_wt) or mutant αA- (αA_144-173) crystallin were further treated with 100 nM STS for 3 h before co-immunoprecipitation with anti-Bax antibody. The precipitated samples were then sequentially probed by western blot using anti-αA/αB and anti-Bax antibodies. IP: immunoprecipitated samples (*left panels*); CE: 20 µg of total proteins from whole cell extract (*right panels*).

We further investigated whether the C-terminal extension domain of αA-crystallin retained its capacity to bind Bax *in vivo*. The interaction of full-length αA-crystallin or αA_144-173 mutant with Bax was assessed by co-immunoprecipitation in 293T cells over-expressing the αA-crystallin constructs and treated for 3 h with 100 nM STS. As observed for full-length αA-crystallin (αA_wt), the C-terminal extension domain (αA_144-173) was sufficient to bind Bax, whereas no immunoprecipitated proteins were observed in cells transfected with the empty vector (pRluc) (Fig. 9).

Altogether these data suggested that the C-terminal extension of αA-crystallin was sufficient to promote the anti-apoptotic action of the protein through binding with Bax and preventing its activation and translocation to the mitochondria.

## Discussion

In the current study, we reported the anti-apoptotic action of αA- and αB-crystallins against Bax-triggered apoptosis. Indeed, caspase-induced apoptosis was inhibited in 293T cells overexpressing α-crystallins, reflected by the inhibition of Caspase-3/-7 activity and the decrease in TUNEL-positive apoptotic cells. By co-immunoprecipitation study, we further showed that αA- and αB-crystallins directly interacted with pro-apoptotic Bax *in vivo*, suggesting that α-crystallins exert their pro-survival action by sequestering Bax in the cytoplasm to prevent its activation and translocation to the mitochondria. In support of this, the overexpressed αA- and αB-crystallins were essentially localized in the cytoplasm of the transfected cells. In lens-derived epithelial cell line, α-crystallins have been shown to inhibit STS-induced apoptosis through interactions with members of the Bcl-2 family. Through binding to Bax and Bcl-X_s_, αA- and αB-crystallins prevented the translocation of the pro-apoptotic proteins from cytosol into mitochondria, repressing the release of cytochrome C and the activation of Caspase-3 upon STS treatment [13]. Pasupuleti et al. demonstrated in HeLa and CHO cells that αA-crystallin inhibited Caspase-9 and Caspase-3 activity and prevented chemically-triggered apoptosis as well as apoptosis induced by over-expression of pro-apoptotic Bim and Bax. In these cells, αA-crystallin-mediated anti-apoptotic function was directly related to its chaperone activity by enhancing PI3K/Akt survival pathway and reducing phosphatase tensin homologue (PTEN) activity [10]. During ER stress-induced retinal pigment epithelial (RPE) cell apoptosis, αB-crystallin has been shown to protect cells against mitochondrial dysfunction by inhibiting Bax and CHOP upregulation, attenuating caspase activation and restoring mitochondrial permeability transition [47]. McGreal and colleagues also reported that αB-crystallin interacted with cytochrome C and preserved mitochondrial membrane potential under oxidative stress [48]. Additionally, in mouse retinal explants exposed to oxidative stress, RPE-secreted αB-crystallin was able to provide neuroprotection to adjacent photoreceptor cells through inhibition of Caspase-3 and PARP activation [49].

Several studies reported on altered expression of α-crystallins in inherited retinal diseases [28], [30], light-induced retinal degeneration [32] as well as early- and late-stage ARMD [33], [50]–[52]. It is thus tempting to speculate that α-crystallins may be involved in the development of these degenerative diseases. However, their role in inherited retinal degeneration has not been studied yet and the molecular mechanisms that may regulate α-crystallin-mediated protection against photoreceptor apoptosis remain unknown. This prompted us to assess the cytoprotective role of α-crystallins in the survival of photoreceptor-like 661W cells. We reported a dose-dependent decrease in cellular viability following STS treatment in lentiviral-mediated 661W cells stably expressing αA- or αB-crystallin, as reflected by increased TUNEL-positive apoptotic cells and decreased cellular ATP content. Moreover, we showed that α-crystallins prevented apoptosis through the inhibition of Caspase-3/-7 activity. It has been shown *in vivo* in αA^−/−^-crystallin and αB^−/−^-crystallin knock-out mice that RPE lacking α-crystallins was more susceptible to apoptosis when subjected to H_2_O_2_-induced oxidative stress, highlighted by increased Caspase-3 activation and elevated mitochondrial permeability transition [53]. Similarly, retinal degeneration induced by CoCl_2_-mediated chemical hypoxia was exacerbated in retina deficient for αA- or αB-crystallin, resulting in earlier and augmented apoptosis in inner and outer nuclear layers and in RPE [34]. αA- and αB-crystallins were described to accumulate in Bruch's membrane-choroid complex in ARMD patients, suggesting that their accumulation reflects disease-related stress response during progression of the disease [33]. Moreover, αB-crystallin displayed a pro-survival effect in RPE in response to Caspase-3-dependent oxidant-mediated apoptotic cell death, suggesting its involvement as a stress-inducible anti-apoptotic protein in the pathogenesis of ARMD [20]. In early experimental autoimmune uveitis (EAU), increased levels of αA-crystallin were reported, while αB-crystallin was not altered [35]. The upregulated αA-crystallin was mostly localized in photoreceptor inner segments that are the site of mitochondrial oxidative stress. αA-crystallin suppressed apoptosis in early EAU through interaction with nitrated Cytochrome c and through inhibition of autoproteolytic maturation of pro-Caspase-3. Increased level of the protein was correlated with protection against photoreceptor cell loss, indicating that αA-crystallin might provide a protective mechanism against immune-mediated mitochondrial oxidative stress-induced photoreceptor apoptosis [35]. A recent study showed that intravenous administration of αA-crystallin prevented photoreceptor apoptosis and degeneration during EAU, whereas αB-crystallin lacked any protective effect [36]. Furthermore, administration of αA-crystallin caused reduced expression of Th1 cytokines as well as Toll-like receptors and their associated adaptators, suggesting that αA-crystallin-mediated protection of photoreceptor loss is associated with systemic suppression of both the adaptive and innate immune response. α-Crystallins have also been reported to exert a neuroprotective effect against retinal ganglion cell (RGC) degeneration. Indeed, intravitreal administration of α-crystallins enhanced survival of axotomized axons [54], while *in vivo* electroporation of αA- and αB-crystallins favored survival of RGCs upon optic nerve injury [55]. Altogether, these data indicate that α-crystallins may trigger common as well as independent intracellular signals and may act either independently or in concert to exert cytoprotective action, depending on the cell type and the disease.

α-Crystallins are constituted of three distinct domains. Each of these domains displays chaperone function which can depend on post-translational modifications of the N-terminus including oxidation, phosphorylation, deamidation, acetylation and truncation [26] [56]. The C-terminal extension is considered to contribute to its chaperone-like activity [57], while the N-terminal domains contain phosphorylation sites that are the targets of various protein kinases [58]. Peptides derived from both αA- and αB-crystallins have been shown to display anti-apoptotic properties in RPE cells upon oxidative stress [59]. However, Santhoshkumar et al. [60] also reported on αA-crystallin-derived peptide accumulating in the aging lens and inhibiting the chaperone activity of α-crystallin. To better understand the protein domain sufficient to provide αA-crystallin-mediated anti-apoptotic effect. we generated various deletion mutants. αA_1-116 and αA_117-173 mutants were chosen because the R116C mutation of αA-crystallin has been shown to reduce its anti-apoptotic activity [13], [14] and to weaken its interaction with Bax [13]. R116C point mutation in αA-crystallin causes autosomal dominant congenital cataract in humans [61]. Mao *et al* [13] previously reported that the N-terminal αA_1-89 mutant did not interact with Bax and did not protect from apoptosis. We refined this analysis and showed that amino acids 90–143 and 144–173 of αA-crystallin were sufficient to protect from Bax-induced apoptosis and were as efficient as the full-length αA-crystallin. We confirmed that N-terminal and showed that α-crystallin domains do not play a major role in the anti-apoptotic properties of αA-crystallin in preventing Bax-induced cell death. Characterization of these mutants allowed us to identify a small αA-crystallin sequence of 29 amino acids corresponding to the C-terminal extension of the protein that retained its ability to bind Bax and to prevent apoptosis.

We previously reported downregulated expression of α-crystallins in correlation with Bax-triggered apoptosis of photoreceptors in *Rpe65^−/−^* mice [37]. In the current study, we further analyzed the pro-survival action of α-crystallins against Bax-induced apoptosis in various cell lines including the photoreceptor-like 661W cells. We demonstrated that α-crystallins exert anti-apoptotic action against Bax-mediated and STS-induced apoptosis and that they act through inhibition of downstream caspases. Furthermore, we reported that the C-terminal extension domain of αA-crystallin was sufficient to provide protection against Bax-triggered apoptosis. However, further studies are needed to address *in vivo* the cytoprotection of α-crystallins against photoreceptor cell death in retinal degeneration, and more specifically to challenge whether they may be efficient to prevent Bax-dependent rod apoptosis in *Rpe65*-related LCA disease. Detailed investigation of α-crystallin-derived peptides may prove to be valuable toward the development of therapeutic molecules for retinal diseases such as inherited retinal degeneration and ARMD.
